# Does the Effect of Micro-Environmental Factors on a Street’s Appeal for Adults’ Bicycle Transport Vary across Different Macro-Environments? An Experimental Study

**DOI:** 10.1371/journal.pone.0136715

**Published:** 2015-08-28

**Authors:** Lieze Mertens, Jelle Van Cauwenberg, Ariane Ghekiere, Veerle Van Holle, Ilse De Bourdeaudhuij, Benedicte Deforche, Jack Nasar, Nico Van de Weghe, Delfien Van Dyck

**Affiliations:** 1 Department of Movement and Sport Sciences, Faculty of Medicine and Health Sciences, Ghent University, Watersportlaan 2, B-9000, Ghent, Belgium; 2 Department of Public Health, Faculty of Medicine and Health Sciences, Ghent University, De Pintelaan 185, 4k3, B-9000, Ghent, Belgium; 3 Department of Human Biometry and Biomechanics, Faculty of Physical Education and Physical Therapy, Vrije Universiteit Brussel, Pleinlaan 2, B-1050, Brussels, Belgium; 4 Research Foundation Flanders (FWO), Egmontstraat 5, 1000, Brussels, Belgium; 5 Ohio State University, City and Regional Planning, 292 Knowlton Hall, West Woodruff Avenue 275, Columbus, OH, 43210, United States of America; 6 Department of Geography, Faculty of Sciences, Ghent University, Krijgslaan 281, S8, B-9000, Ghent, Belgium; University of Missouri, UNITED STATES

## Abstract

**Background:**

Characteristics of the physical environment can be classified into two broad categories: macro- (“raw” urban planning features influenced on a regional level) and micro- (features specifically within a streetscape influenced on a neighborhood level) environmental factors. In urban planning applications, it is more feasible to modify conditions at the neighborhood level than at the regional level. Yet for the promotion of bicycle transport we need to know whether relationships between micro-environmental factors and bicycle transport depend on different types of macro-environments. This study aimed to identify whether the effect of three micro-environmental factors (i.e., evenness of the cycle path surface, speed limits and type of separation between cycle path and motorized traffic) on the street’s appeal for adults’ bicycle transport varied across three different macro-environments (i.e., low, medium and high residential density street).

**Methods:**

In total, 389 middle-aged adults completed a web-based questionnaire consisting of socio-demographic characteristics and a series of choice tasks with manipulated photographs, depicting two possible routes to cycle along. Conjoint analysis was used to analyze the data.

**Results:**

Although the magnitude of the overall effects differed, in each macro-environment (i.e., low, medium and high residential density), middle-aged adults preferred a speed limit of 30 km/h, an even cycle path surface and a hedge as separation between motorized traffic and the cycle path compared to a speed limit of 50 or 70 km/h, a slightly uneven or uneven cycle path surface and a curb as separation or no separation between motorized traffic and the cycle path.

**Conclusions:**

Our results suggest that irrespective of the macro-environment, the same micro-environmental factors are preferred in middle-aged adults concerning the street’s appeal for bicycle transport. The controlled environment simulations in the experimental choice task have the potential to inform real life environmental interventions and suggest that micro-environmental changes can have similar results in different macro-environments.

## Background

Globally, 31% of adults aged 15 years or older are insufficiently physically active, [1,2], which is reflected in the rapidly increasing prevalence of inactivity-related health problems [2]. In the European Union, there is a significant unfulfilled potential to increase bicycling behavior of the population, since 50% of all trips are shorter than 3 kilometers, a distance which can be cycled in 10 minutes [3]. In Flanders (Belgium), more than 70% of these trips (≤ 3 km) are currently done using passive transport [4]. Bicycle transport is an accessible, economic, social, and environmentally sustainable form of physical activity, easy to integrate into adults’ daily routines and moreover has the potential to increase physical activity levels in European adults. Cross-sectional studies indicated that bicycle transport is associated with higher general physical activity levels and lower body weight in adults [5]. Prospective observational studies demonstrated a strong inverse relationship between bicycle transport and all-cause mortality, cancer mortality, and cancer morbidity among middle-aged participants [6]. In addition, active transport has many other positive economic, social, environmental and traffic management effects [6–15]. Because of these benefits, communities should encourage people to cycle on a regular or daily basis [16–19]. One long-term approach to do this involves changes to the physical environment to make it more supportive of bicycling [20]. Environmental interventions and policies targeting the physical environment, can reach large populations over long periods of time and encourage more bicycling and less reliance on the car [7,21,22]. The physical environment can be defined as “the objective and perceived characteristics of the physical context in which people spend their time (e.g. home, neighborhood), including aspects of urban design, traffic density and speed, distance to and design of venues for physical activity (e.g., parks), crime and safety” [23]. Previous studies already indicated that the physical environment appears to be an important contributor to encourage bicycle transport among middle-aged adults [22,24].

Characteristics of the physical environment can be classified into two broad categories: macro- and micro-environmental factors [25,26]. Macro-environmental factors can be defined as the more “raw” urban planning features; such as street network density, residential density and land use diversity. These factors may be difficult to change in existing neighborhoods, because of their size and complexity and moreover because this would require a strong collaboration between regional authorities. Macro-environmental factors are essentially beyond the influence of individuals and even for governments and nongovernmental organizations it is usually difficult to modify large existing structural features [25,26]. Micro-environmental factors, however, can be defined as specific characteristics of environmental features within a streetscape; such as evenness cycle path surface, vegetation and speed limits. In urban planning applications, it is more feasible to modify conditions at the neighborhood level than at the regional level because these micro-environmental factors are relatively small-scaled environmental factors and potentially influenced by individuals or local actors [25,26]. Therefore, the reconfiguration of micro-environmental factors in existing neighborhoods involves a lower cost and a shorter time-frame compared to the reconfiguration of the macro-scale structural design [25,26], making micro-environmental factors more practical and promising to target in physical environmental interventions of existing neighborhoods. Studies around the world have found consistent positive relationships between macro-environmental factors (including walkability, access to shops/services/work, and urbanization) and transport-related bicycling in adults [25,26]. Unfortunately, the relationships between bicycle transport and more amenable, micro-environmental factors are less consistent [27–29]. These inconsistencies in the literature are potentially attributed to the used methodology.

Although various studies have investigated the relationships between the physical environment and physical activity, they are often cross-sectional and thus not able to establish causality [30–32]. In these studies, environmental perceptions were generally assessed with questionnaires, which involves some difficulties. First, participants have to recall features of the physical environment, which leads to recall bias [33], and second the lack of standardization in neighborhood definitions increases the inconsistency as well [34]. Moreover, because many physical environmental factors are interrelated in real life conditions, these studies cannot clearly identify the critical environmental correlates of bicycle transport. Experimental studies are required to decrease these inconsistencies and to make causal statements [22,30–32,35]. Since experiments are complex, time- and cost-consuming to conduct in real environments, an innovative experimental and cost-effective methodology is required.

Therefore, the present study opts for a controlled experiment: it uses controlled manipulations of environmental characteristics in photographs to experimentally find out whether these characteristics affect the street’s appeal for bicycle transport. As research shows that responses to photos generalize well to on-site response [36,37], the findings can provide guidelines for interventions that modify micro-environmental factors to increase the street’s appeal for bicycle transport. This methodology was used in two recent mixed-methods studies to determine possible causal relationships between a limited number of key micro-environmental factors and the street’s appeal walking transport among older adults [38] and bicycle transport among adults [39]. This latter pilot study provided a first indication of the effects of changing micro-environmental factors on the street’s appeal for bicycle transport in adults. However, this pilot study had an important limitation: it used only one macro-environment, a typical street environment in a semi-urban (300–600 inhabitants/km^2^) Belgian municipality [40]. For interventions, it is essential to know how well the findings can be generalized to other macro-environments. If micro- and macro- environmental factors are interacting, interventions focusing on micro-environmental factors may have to differ depending on the macro-environment.

Therefore, the current study aims to find out if the effect of manipulated micro-environmental factors (evenness of the cycle path surface, speed limit and type of separation between cycle path and motorized traffic) on the street’s appeal for middle-aged adults’ bicycle transport depends on macro-environmental factors or is generalizable to different macro-environments (i.e. low, medium and high residential density street).

## Methods

### Protocol and measures

Flemish middle-aged adults between 45 and 65 years were recruited by purposeful convenience sampling [41] using email, social media, family, friends, clubs and organizations. By snowball sampling [41], additional participants were recruited. This age group was chosen as adults in this age range do assess the environment according to themselves, rather than in the viewpoint of their children. Participants completed a two-part web-based questionnaire, which was developed using Sawtooth Software (SSIWebversion 8.2.4.). It first assessed socio-demographic characteristics, and second the participant had to perform a series of choice tasks with manipulated photographs (a detailed description of these choice tasks appears later in this paper). Informed consent was automatically obtained from the participants when they voluntarily completed the questionnaire. The online questionnaire was available from the beginning of February until the end of March 2014 and 389 middle-aged adults participated in the study. The study was approved by the Ethics Committee of the Ghent University Hospital.

#### Photograph development

**Fig 1:**
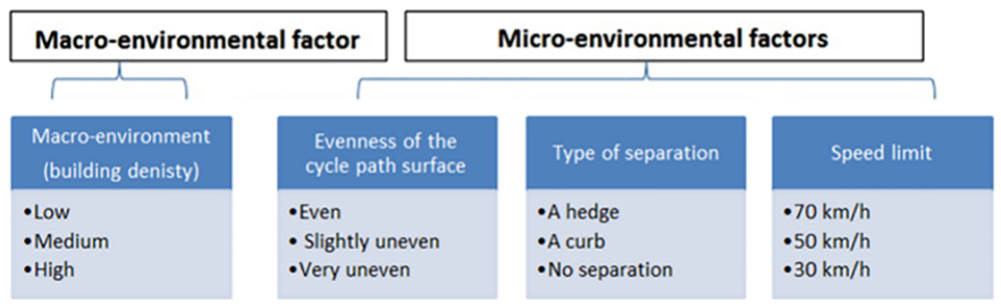
An overview of the environmental factors and their respective levels used in the choice tasks (ref. S1 File).

The manipulated photographs, depicting a possible route to cycle along, used in the choice task were developed with Adobe Photoshop software [42]. Previous research has established the validity of responses to color photos in comparison to on-site responses [36,37]. In each photograph, four environmental factors (one macro- and three micro-environmental factors) were manipulated and each environmental factor consisted of three possible levels (see Fig 1), yielding a total of 81 (= 3^4^) photographs. One of the four manipulated environmental factors was considered as a macro-environmental factor and was defined in this study according to residential building density and land-use mix depicting the general street setting. Three levels were distinguished: (1) environment with low building density (open environment) and single land use, (2) environment with medium building density and single land use, (3) environment with high building density and mixed land use. See S1 File for an example of all three different street settings or macro-environments used in this study. Additionally, three micro-environmental factors (evenness cycle path surface, speed limit and type of separation between cycle path and motorized traffic) were manipulated in each photograph and consisted of an anticipated attractive, intermediate and unattractive level (e.g. even cycle path surface, slightly uneven cycle path surface and a very uneven cycle path surface). These three micro-environmental factors were chosen based on previous research [27,39,43,44] and existing literature on the relationship between the environment and bicycle transport [27,45]. Each photograph was developed from an adult cyclist’s eye level viewpoint, under dry weather conditions and without people visible in the environment. Fig 1 and S1 File provides an overview and illustration of the four manipulated environmental factors with their respective levels (the terms presented in Fig 1 are used throughout the article).

#### The web-based questionnaire

The web-based questionnaire had two parts. The first part assessed socio-demographic characteristics: gender, age, country of birth, education, occupational status, marital status and place of residence (see Table 1 for the response categories). Next, the long form of the International Physical Activity Questionnaire (IPAQ: ‘usual week’) [46] was used to establish the amount of usual amount of bicycle transport in a week. The second part of the questionnaire consisted of a series of choice tasks, based on a choice based conjoint (CBC) method. This CBC method is mainly used in marketing research and enables examination of preferences for various components of a product in the decision process to pursuit the product [47]. In this study the various components are the different manipulated environmental factors and the product is a street’s appeal for bicycle transport along the depicted environments. Despite conjoint analysis is more than forty years old, it continues to evolve by new technology and methodologies [47]. Furthermore, besides marketing research an ongoing stream of research is making use of this technique [48]. Conjoint analysis has been proved to be one of the best tools available for determining relative importance of factors of complex environments from the user point of view [49,50]. Using photographs to display alternatives of complex environments rather than written descriptions, immediately gives a clear view or understanding of what should be assessed (i.e. reduce recall bias). The following scenario was presented to the respondents: “*Imagine yourself bicycling to a friend’s home*, *located at 10 minutes bicycling from your home*, *during daytime with perfect weather circumstances*. *For every task you will see two streets*, *we ask you to choose the street that you find most appeal to cycle along to a friend*. *Whichever route you choose the distance to your friend is the same and all cycle paths are one-way*. *There is no right or wrong solution*, *we are only interested in which street you would prefer to cycle along*.” First, participants could see three examples and afterwards they received a set of 14 randomly assigned choice tasks. More than 20 choice tasks may make respondents less likely to complete the task [51]. The computer program randomly determined the picture pairs that appeared in the choice tasks, allowing each level within each attribute to appear an equal number of times in the choice task and consequently allowing that not all possible combinations need to be presented to each participant.

**Table 1:** Descriptive characteristics of the participants (n = 389).

**Age (M ± SD) (year)**	53.8 ± 5.2	**Occupational status (%)**	
**Women (%)**	55.3	- Household	4.4
**Born in Belgium (%)**	96.4	- Blue collar	6.7
**Marital status (%)**		- White collar	68.9
- Married	75.3	- Unemployed	3.9
- Widowed	2.3	- Retired	15.2
- Divorced	10	- Career interruption	1
- Single	4.4	**BMI (M ± SD)**	24.8 ± 4.0
- Cohabiting	8	**Current bicycle transport level**	
**Education (%)**		- Bicycle transport min/wk (M ± SD)	37.3 ± 33.1
- Primary	1.8	- No bicycling for transport (%)	24.69
- Lower secondary	19		
- Higher secondary	13.4		
- Tertiary	65.8		

M = mean; SD = standard deviation; BMI = body mass index

An a priori power analysis (power 0.80 and α = 0.05) calculated by the following formula: *nta /c > 500 (n = number of participants; t = 14*: *number of choice tasks; a = 2*: *number of alternatives per task; c = 9*: *the largest product of levels of any two factors*) [47] showed that a minimum of 161 subjects was needed when manipulating four environmental factors in one photograph (with three levels each) and presenting 14 choice tasks to each participant.

To assess test-retest reliability, we conducted a pilot study (n = 27) in which four fixed tasks were deliberately added to the set of choice tasks. In this pilot study, participants had to complete 16 choice tasks. The same two choice tasks were presented at the beginning and at the end of the questionnaire. These choice based conjoint tasks were identical for all participants (= fixed tasks). Subsequently, it was examined whether participants chose the same street both times. The percentage of agreement for the first task was 81% and 93% for the second fixed task (n = 27). An adequate level of agreement is generally considered to be 70% [52]. These results indicated that our choice tasks are reliable.

### Analyses

#### Choice-based conjoint analysis (CBC)

Choice-based conjoint analysis (CBC) was used to analyze the data [47]. First, average part-worth utilities were calculated from the individual utilities gained from hierarchical Bayes (HB) estimation to determine the main effects of each environmental factor on the street’s appeal for bicycle transport along the depicted environments. This has been suggested as the most appropriate method to analyze data gained from choice based conjoint [53]. Utilities represent the degree of preference given to a particular level of an environmental factor and are similar to a β obtained from linear regression analyses [47].

Second, the average relative importance of each environmental factor was calculated from the individual utility data gained from hierarchical Bayes (HB) estimation. Utility values cannot be compared across components, because they have different metrics, but each component has a unique scale determined through the hierarchical Bayes estimation procedure. Therefore, the difference in individual utilities between the most and least preferred levels of a component can be used to represent the importance of each component for each respondent [47]. This individual importance represents the relative importance of each environmental factor when judging a street on the street’s appeal for adults’ bicycle transport. An individual importance is calculated by subtracting the lowest from the highest utility for the given factor and dividing this by the sum of differences across all components for that participant. The individual relative importance can in turn be used to calculate the average relative importance of each component for the total sample [47]. Furthermore, also the relative importance of the micro-environmental factors within each macro-environment was calculated.

Third, interaction effects were also derived from part-worth utilities gained from the hierarchical Bayes (HB) estimation using dummy coding (burn in: 100,000; total iterations: 1,100,000). Three separate models were constructed to analyze the interaction effects of micro-environmental factors with the macro-environment: ‘macro-environment by evenness of the cycle path surface’, ‘macro-environment by speed limit’ and ‘macro-environment by type of separation between cycle path and motorized traffic’. The interaction effects were illustrated by graphs and tables in which the total utilities of the different streets were shown. Total utilities were calculated by the sum of the part-worth utilities, representing the degree of preference given to a particular level of an environmental factor. The size of the interaction effect was determined by calculating the difference in total utilities for each participant separately. The average of these values over all subjects, is the average interaction effect. A 95% confidence interval was calculated to define significance.

## Results

### Descriptive statistics

In total, 389 adults (214 women and 175 men) between 45 and 65 years participated in the study. Most participants (65.8%) reported a tertiary education degree (higher, university or postgraduate). Approximately one quarter of the participants said they did not cycle for transport in a usual week. See Table 1 for other descriptive characteristics of the sample.

### Main effects of the environmental factors

For the macro-environmental factor, participants preferred a low (average part-worth utility = 1.93±3.45; 95% CI: 1.59, 2.27) residential density street above a medium (0.76±1.89; 95% CI: 0.58, 0.95) or high (reference level) residential street. Moreover, they preferred a medium residential density street to a high residential street. For the micro-environmental factors, participants preferred an even cycle path surface (3.61±5.01; 95% CI: 3.11, 4.11) to a slightly uneven (2.25±2.49; 95% CI: 2.00, 2.50) or very uneven (reference level) cycle path; and they preferred a slightly uneven cycle path surface to a very uneven cycle path surface. They also preferred a traffic limitation of 30 km/h (4.43±2.90; 95% CI: 4.14, 4.72) to one of 50 km/h (2.51±1.62; 95% CI: 2.35, 2.67) or 70 km/h (reference level) and they preferred a traffic limitation of 50 km/h to one of 70 km/h. Finally, the participants preferred a cycle path separated from traffic with a hedge (4.91±3.13; 95% CI: 4.60, 5.22) to one separated from traffic with a curb (2.04±1.90; 95% CI: 1.86, 2.23) or one located on the street (reference level), and they preferred a cycle path separated from traffic with a curb to one located on the street. See S1 File for an illustration of the different manipulations (e.g. the different types of separations between cycle path and motorized traffic).

### Relative importance of the environmental factors

The average importance of the four factors, based on individual utility calculations, shows that the macro-environment was the least important factor in making choices among the different street alternatives. Given that there is no overlap between the confidence intervals of the micro-environmental factors, it appears that the average importance of the macro-environment (18.15±14.66%; 95% CI: 16.69, 19.61) was significantly lower compared to the three micro-environmental factors. The three micro-environmental factors, however, did not significantly differ from each other in relative importance with 26.71±22.36% (95% CI: 24.48, 28.93) for evenness of the cycle path surface, 26.68±16.50% (95% CI: 27.04, 30.32) for type of separation between cycle path and motorized traffic and 26.47±15.62% (95% CI: 24.92, 28.02) for speed limit.

### Relative importance of the micro-environmental factors within each macro-environment

**Fig 2:**
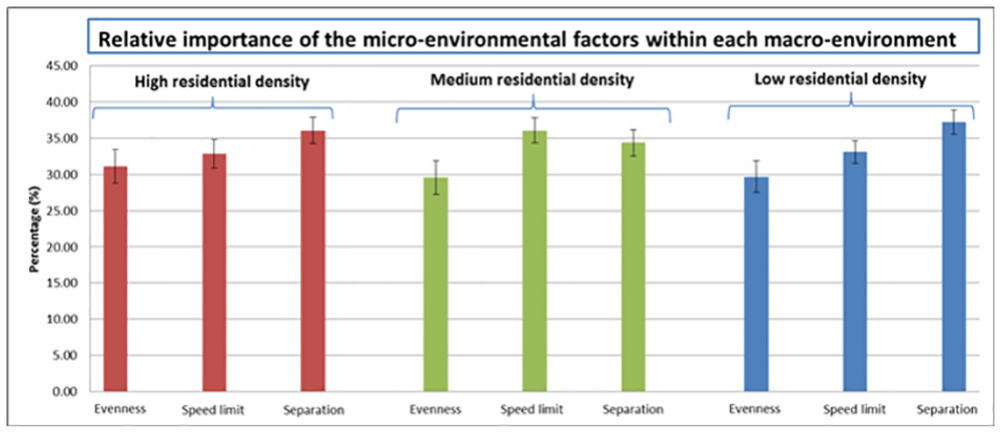
The average relative importance of the three micro-environmental factors in each macro-environment.

Greater importance was found for type of separation between cycle path and motorized traffic than for evenness of the cycle path surface in each macro-environment (see Fig 2). The results showed that in a low residential density environment a separation (37.20±16.98%; 95% CI: 35.51, 38.89) was also more important than the presence of speed limit (33.10±15.47%; 95% CI: 31.56, 34.63). In a medium residential density environment, the presence of a speed limit (36.05±17.56%; 95% CI: 34.31, 37.80) was more important than the evenness of the cycle path surface (29.57±23.13%; 95% CI: 27.27, 31.87). The remaining importance of the micro-environmental factors did not significantly differ from each other in each macro-environment.

### Interaction effects

#### Interaction between macro-environment and evenness of the cycle path surface

**Fig 3:**
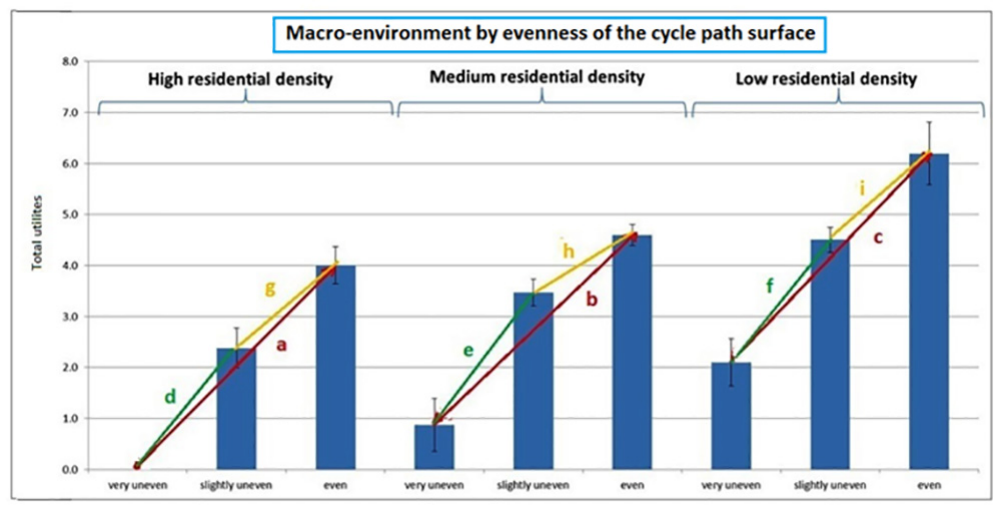
Interaction effect between the macro-environment and the evenness of the cycle path surface. Note: a, b, c, d, e, f, g, h, i = the distance between the total utilities; * = p<0.05; a > b*, a < c, b < c*, d < e*, d < f, e > f*, g < h*, g < i, h < i*.

Fig 3 shows the overall utilities for streets differing in macro-environment and evenness of the cycle path surface. The characters on Fig 3 illustrate the distance between the total utilities. In each macro-environment, an even cycle path surface was preferred for bicycling to a slightly or very uneven cycle path surface, and a slightly uneven was preferred to a very uneven cycle path surface. Only the strength of this positive effect of evenness of the cycle path surface differed between the three macro-environments. The effect of an even cycle path surface compared to a very uneven cycle path surface or a slightly uneven cycle path surface was lower in a medium residential density environment compared to a high or low residential building density (see Table 2 and Fig 3). No significant difference in effect of evenness of the cycle path surface emerged for comparisons of a low to a high residential density environment.

**Table 2:** Interaction effect between the macro-environment and evenness of the cycle path surface, speed limit and type of separation between cycle path and motorized traffic.

**Macro-environment and evenness of the cycle path surface**									
**Evenness**	***Very uneven—even***			***Very uneven—slightly uneven***			***Even—slightly uneven***		
**Macro**	***High—Med***	***High—Low***	***Med—Low***	***High—Med***	***High—Low***	***Med—Low***	***High—Med***	***High—Low***	***Med—Low***
**MEAN**	-0.28	0.09	0.37	0.21	0.02	-0.19	-0,49	0,07	0,57
**SD**	0.93	1.53	1.10	1.06	1.08	1.44	0,95	1,15	1,03
**-95% CI**	-0.37	-0.06	0.27	0.11	-0.09	-0.33	-0,59	-0,04	0,46
**+95% CI**	-0.19	0.25	0.48	0.32	0.13	-0.05	-0,40	0,19	0,67
Fig 3	a > b*	a < c	b < c*	d < e*	d < f	e > f*	g < h*	g < i	h < i*
**Macro-environment and speed limit**									
**Speed limit**	***70 km/h—30 km/h***			***70 km/h—50 km/h***			***30 km/h—50 km/h***		
**Macro**	***High—Med***	***High—Low***	***Med—Low***	***High—Med***	***High—Low***	***Med—Low***	***High—Med***	***High—Low***	***Med—Low***
**MEAN**	-0,62	0,19	0,81	-4,19	0,11	4,30	3,57	0,08	-3,49
**SD**	0,96	0,91	1,21	1,94	1,24	1,80	1,96	0,58	2,03
**-95% CI**	-0,72	0,10	0,69	-4,38	-0,02	4,12	3,37	0,02	-3,69
**+95% CI**	-0,53	0,28	0,93	-4,00	0,23	4,47	3,76	0,14	-3,28
Fig 4	j > k*	j < l*	k < l*	m > n*	m < o	n < o*	p > q*	p < r*	q > r*
**Macro-environment and type of separation between cycle path and motorized traffic**									
**Type of separation**	***No separation—hedge***			***No separation—curbe***			***Hedge—curb***		
**Macro**	***High—Med***	***High—Low***	***Med—Low***	***High—Med***	***High—Low***	***Med—Low***	***High—Med***	***High—Low***	***Med—Low***
**MEAN**	-0.25	-0.10	0.15	0.85	0.46	-0.38	-1.09	-0.56	0.54
**SD**	1.43	2.11	1.59	1.41	1.71	2.55	0.99	1.66	1.63
**-95% CI**	-0.39	-0.30	-0.01	0.71	0.29	-0.64	-1.19	-0.72	0.37
**+95% CI**	-0.10	0.11	0.31	0.99	0.63	-0.13	-0.99	-0.39	0.70
Fig 5	s > t*	s > u	t < u	v < w*	v < x*	w > x*	y > z_1_ *	y < z_²_ *	z_1_ < z_2_ *

RSD = residential building density, SD = standard deviation, CI = confidence interval,

* = p<0.05

**Fig 4:**
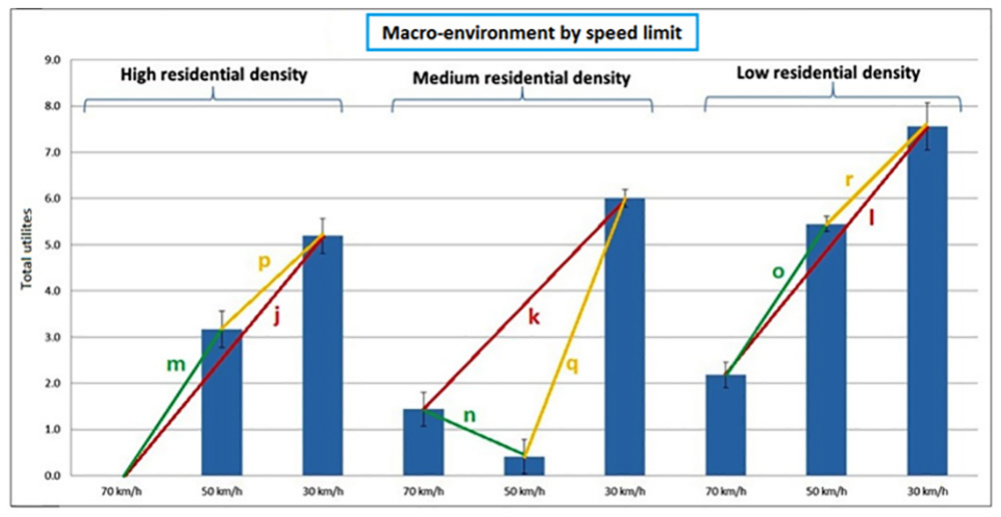
Interaction effect between the macro-environment and speed limit. Note: j, k, l, m, n, o, p, q, r = the distance between the total utilities; * = p<0.05; j > k*, j < l*, k < l*, m > n*, m < o, n < o*, p > q*, p < r*, q > r*.

**Fig 5:**
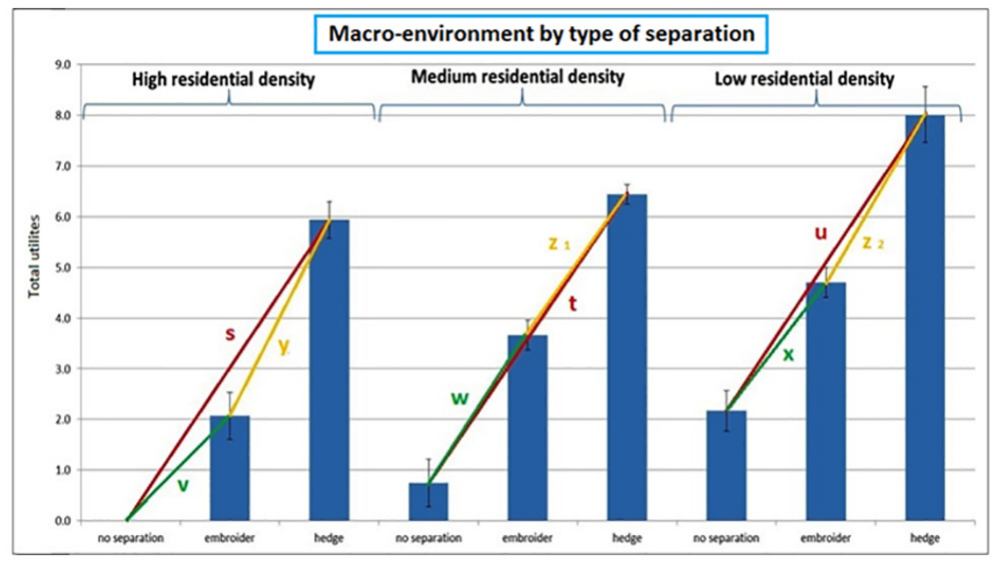
Interaction effect between the macro-environment and type of separation between cycle path and motorized traffic. Note: s, t, u, v, w, x, y, z^1^, z^2^ = the distance between the total utilities; * = p<0.05; s > t*, s > u, t < u, v < w*, v < x*, w > x*, y > z1*, y < z2*, z1 < z2*.

Note: a, b, c, d, e, f, g, h, i, j, k, l, m, n, o, p, q, r, s, t, u, v, w, x, y, z^1^, z^2^ = the distance between the total utilities, which are marked on Figs 3, 4 and 5.

#### Interaction between macro-environment and speed limit

Fig 4 shows the overall utilities for streets differing in macro-environment and speed limit. In each macro-environment the most strict speed limit (30 km/h) was preferred first and secondly 50 km/h above 70 km/h. Only in a medium residential density environment the participants preferred a speed limit of 70 km/h above 50 km/h. The positive effect of a speed limit of 30 km/h compared to 70 km/h or 50 km/h was largest in a low residential density environment, except for the effect of a speed limit of 30 km/h compared to 50 km/h which was stronger in a medium residential density environment (see Table 2 and Fig 4).

#### Interaction between macro-environment and type of separation between cycle path and motorized traffic

Fig 5 shows the overall utilities for streets differing in macro-environment and type of separation between cycle path and motorized traffic. In each macro-environment, participants preferred first a hedge as separation between cycle path and motorized traffic and secondly a curb above a cycle path located on the street. However, the strength of the effect of the type of separation differed between the different macro-environments. The effect of a hedge instead of no separation was larger in a high compared to a medium residential density environment. There was no significant difference of this effect between a high compared to low and a low compared to medium residential density environment. Moreover the effect of a curb instead of no separation was largest in a medium and also greater in a low compared to high residential density environment (see Table 2 and Fig 5).

## Discussion

This is the first controlled experiment to examine the effect of manipulating micro-environmental factors on the environment’s perceived street’s appeal for adults’ bicycle transport in different street settings or macro-environments. The analyses indicate that the effect of micro-environmental modifications may well generalize to physical environmental interventions in different macro-environments. Although we found several statistically significant interactions between the micro-environmental factors and the macro-environmental factor, the direction of the effects across the different macro-environments did not differ, only the magnitude of the effect did.

There was no significant difference in relative importance between the three micro-environmental factors, independent of the macro-environment. Thus, all three micro-environmental factors were equally important for the street’s appeal for bicycle transport. However, within each macro-environment, small differences in relative importance of the micro-environmental factors were detected. The presence of a separation was more important than evenness of the cycle path surface in all three macro-environments. In a low residential density environment, a separation was also more important than a speed limit and in a medium residential density environment, the presence of a speed limit was more important than evenness of the cycle path surface. Otherwise, the relative importance of the three micro-environmental factors was similar in each macro-environment. Since the real environment consists of a large number of micro-environmental factors, it is essential for future research to include all possible environmental factors in photograph experiments in order to make the best simulation of the real environment. However, this research step was necessary because it would be unmanageable to manipulate all possible micro-environmental factors, together with different macro-environmental factors in the photographs.

The current study also showed that the macro-environment was less important for the street’s appeal for bicycle transport than the three micro-environmental factors. This suggests that each improvement in a micro-environmental factor is a promising practical direction for interventions. However, our finding that participants preferred a low to either a medium or high residential density environment for bicycle transport differs from previous cross-sectional studies. Those studies indicated that certain macro-environment factors, including walkability, access to shops/services/work and degree of urbanization with bicycle transport, are associated with more bicycle transport [27]. People living in more urbanized areas or in a highly walkable environment tend to do more bicycle transport than people living in less walkable environments [27,54,55]. Although a more walkable environment probably encourages bicycling through the short access to shops/services/work, the current study shows that the view of a less walkable environment is more appealing for bicycling. It is possible that these contrasting findings result from the standardized 10 minute travel time in the present study. Concerning walkability, the distance to a destination is a crucial aspect for transportation behavior and in low dense areas, travel distances are usually larger than 10 minutes. Adding the effect of distance as an additional factor to the choice tasks, could be a good research question for future research. The discrepancy between our finding and previous findings may also result from differences between the perception of the street’s appeal for bicycle transport, the intention to cycle and the actual bicycling behavior [56,57].

Examination of the importance of the levels within each environmental factor (main effects) revealed that an even cycle path surface, a speed limit of 30 km/h, and a hedge between cycle path and motorized traffic were most preferred to middle-aged adults for the street’s appeal for bicycle transport along the depicted environments. A previous study using manipulated photographs yielded similar results [39]. The present study confirmed these results but for different macro-environments, which was the ultimate goal in this research. In addition, previous cross-sectional studies found evenness of the cycle path surface [58,59], speed limit [45,60] and type of separation between cycle path and motorized traffic [59,61,62] to be related to bicycle transport. Our results indicated that small changes in the micro-environmental factors can help to increase the street’s appeal for bicycle transport in various macro-environments. Since in this study only a selection of three micro-environmental factors and not all potentially relevant micro-environmental factors (e.g., general upkeep of the environment, presence of vegetation, traffic volume) were used in these experiments, further research is needed to determine the effect of all those factors.

The main strength of this study is the used innovative methodology that enables to establish causal relationships between environmental manipulations and the street’s appeal for bicycle transport. Because it is very difficult and expensive to investigate the effects of changing real environments on actual bicycling behavior, this study used a cost-effective approach by manipulating photographs of environments. However, further research in real-life settings is warranted to find out whether current findings can be replicated when studying the effects of real environmental modifications on actual bicycling behavior. Furthermore, these causal relationships cannot be recorded by more recent methodologies like photo-elicitation [63] (requiring participants to take photo images of their journey and revealing actual preferences) or GPS [64] (indicates where an individual is active). These methodologies could help to record changes in behavior as a result of natural experiments, but could not give information about the importance of micro- and macro-environmental factors. The current study adds to the literature, as it is still unclear what type of infrastructure is required to encourage bicycle transport. This might be due to the fact that environmental factors have not been specified enough to elicit associations between bicycle transport and built environment in previous studies [65]. In contrast, our study focuses on small, amenable micro-environmental factors, which are feasible to modify during interventions. Furthermore, it remains important to keep in mind that interventions should not focus on only one particular determinant of active transport, such as the built environment. Evidence shows that multi-layered interventions are most successful to initiate and sustain behavior change effectively [7,65].

Future research can benefit from some of the strengths of the present approach: the use of different macro-environments to examine the effect of manipulating micro-environmental factors, testing responses to photographs rather than verbal descriptions of places, the use of manipulated simulations to create a controlled experiment, and the use of a choice-based conjoint method (CBC), which allow testing for effects without presenting all of the possible combinations. The study also allowed to test more items that were combined at the same time. Such methodology can answer questions about effects of environmental changes on the street’s appeal for adults’ bicycle transport. The same method can also be used for different subgroups like children and senior citizens, and perhaps can also be used for other behaviors such as walking or general physical activity. Consequently, these controlled simulations can provide ready-made advice for natural experiments, which can be considered a logical next step in this study project. Findings obtained from research using manipulated photographs could inform physical environmental interventions in real life settings about which environmental factors to modify in different macro-environments.

There are, however, some limitations that have to be acknowledged. First, the present study assessed effects on the street’s appeal for bicycle transport and, not actual bicycling behavior. Consequently, studies are needed to examine the effects of changing real environments on bicycling behavior in various contexts. Second, the present study focused on three micro-environmental factors. Although previous research had indicated them as important factors, perhaps other factors would have effects alone or in interaction with other factors. Future research should identify and include all potentially relevant micro-environmental factors and investigate their interactions. Third, a limitation of using color photographs is the two-dimensional character or the lack of movement in the environment. In real life, people notice different things in the environment depending on their speed of travel. Manipulating computer-generated virtual walkthrough environments (three-dimensional) could offer a solution for this problem [66]. Fourth, our sampling yielded a sample of well-educated adults with 65.8% having a tertiary education degree. This is much more than the statistics for the Flemish population indicate with 28.1% having a tertiary degree [67]. Future research needs to establish how well the findings apply to other less educated groups. A study from Scheepers et al. (2013) [68] indicated a higher use of active transport modes by persons with an university or college degree. Because there are differences in bicycling behaviors between individuals with a different individual educational level, future research should also investigate the moderating effects of other personal determinants (such as gender, age and employment) on the relationships between manipulating the environment and the street’s appeal for bicycle transport as well [22].

## Conclusions

The present study used different macro-environments to examine the effect of manipulating micro-environmental factors. Our findings indicate that in each different macro-environment (i.e. low, medium and high residential density), middle-aged adults preferred a speed limit of 30 km/h, an even cycle path and a hedge as separation between motorized traffic and the cycle path compared to a speed limit of 50 or 70 km/h, a slightly uneven or uneven cycle path surface and a curb as separation or no separation between motorized traffic and the cycle path. The direction of these effects were all the same in each macro-environment, only the magnitude of the effects differed between the different macro-environments. Our results suggest that irrespective of the macro-environment, the same micro-environmental factors are preferred in middle-aged adults concerning the street’s appeal for bicycle transport. Consequently, no other physical environmental factors might be modified in different street settings. Any small changes to the micro-environmental factors (e.g. changing the speed limits from 50 km/h to 30 km/h) can effectively help to increase the street’s appeal for bicycle transport among adults. These controlled simulations could inform environmental interventions in real life settings to modify similar micro-environmental factors in different macro-environments. However, these findings need to be confirmed by on-site research.

## Supporting Information

S1 File (PDF)
