# Supplementary figures and images for: Does the Effect of Micro-Environmental Factors on a Street’s Appeal for Adults’ Bicycle Transport Vary across Different Macro-Environments? An Experimental Study
Source: PLoS One. 2015 Aug 28;10(8):e0136715. doi: 10.1371/journal.pone.0136715 (PMC4552783; doi:10.1371/journal.pone.0136715)

## S1 File

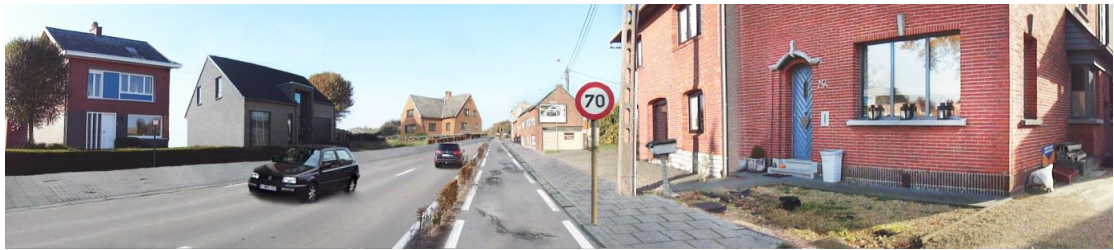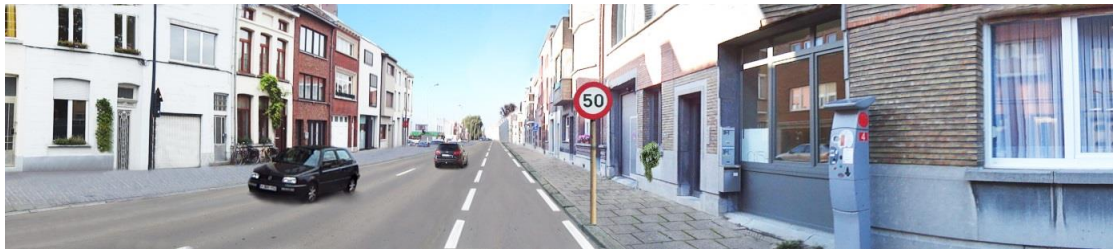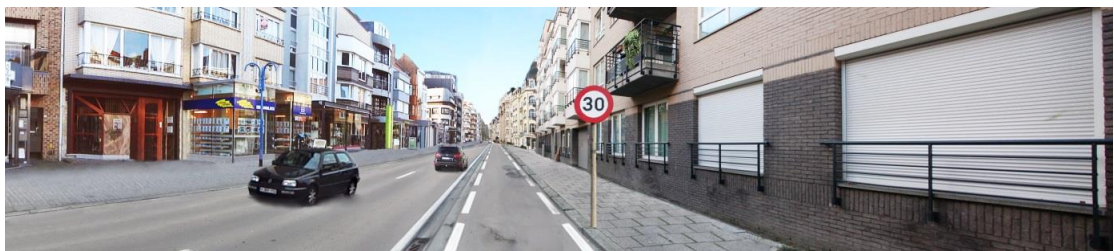

Supplement: S1 File — (PDF) [file pone.0136715.s001.pdf]
